# Metastability of Synchronous and Asynchronous Dynamics

**DOI:** 10.3390/e24040450

**Published:** 2022-03-24

**Authors:** Emilio Nicola Maria Cirillo, Vanessa Jacquier, Cristian Spitoni

**Affiliations:** 1 Dipartimento di Scienze di Base e Applicate per l’Ingegneria, Sapienza Università di Roma, Via A. Scarpa 16, 00161 Roma, Italy; 2 Dipartimento di Matematica e Informatica “Ulisse Dini”, Università degli Studi di Firenze, Viale Morgagni 67/a, 50134 Firenze, Italy; vanessa.jacquier@unifi.it; 3 Institute of Mathematics, University of Utrecht, Budapestlaan 6, 3584 CD Utrecht, The Netherlands; c.spitoni@uu.nl

**Keywords:** metastability, lattice spin systems, probabilistic cellular automata, synchronous dynamics, asynchronous dynamics

## Abstract

Metastability is a ubiquitous phenomenon in nature, which interests several fields of natural sciences. Since metastability is a genuine non-equilibrium phenomenon, its description in the framework of thermodynamics and statistical mechanics has progressed slowly for a long time. Since the publication of the first seminal paper in which the metastable behavior of the mean field Curie–Weiss model was approached by means of stochastic techniques, this topic has been largely studied by the scientific community. Several papers and books have been published in which many different spin models were studied and different approaches were developed. In this review, we focus on the comparison between the metastable behavior of synchronous and asynchronous dynamics, namely, stochastic processes in discrete time in which, at each time, either all the spins or one single spin is updated. In particular, we discuss how two different stochastic implementations of the very same Hamiltonian give rise to different metastable behaviors.

## 1. Introduction

Metastable states are commonly observed in several diverse fields, such as physics, chemistry, biology, computer science, climatology, and economics. In order to portray their main features we use, here, the classical example of super-saturated vapors: under special experimental conditions, a vapor can be compressed at pressures lower than the value at which liquefaction should start. The vapor, thus, enters a state that is different from the thermodynamic equilibrium phase, but, for small variations of the thermodynamics parameters, it behaves as if it were in real equilibrium following the laws of thermodynamics and undergoing small reversible changes. The vapor can remain in such a state for a very long time, but it can exit such a state and reach the real thermodynamic phase (the liquid phase) via internal random fluctuation or external perturbations [1]. The eventual transition from the vapor to the liquid phase is irreversible.

Other well-known contexts in which metastability is observed are the crystallization of proteins and ferromagnetic materials (branches of the hysteresis loop, where the magnetization is opposite to the external magnetic field).

The study of metastability has attracted much attention in the last decades not only for its intrinsic interest, but also because metastable states are an example of non-equilibrium states of thermodynamical systems. As it is well known, statistical mechanics has developed a complete mathematical formalism, even on a rigorous basis [2], to describe equilibrium states, whereas a complete consistent theory of systems out of equilibrium is still lacking. Thus, deriving a rigorous mathematical theory of metastable states can shed same light on rigorous approaches to non-equilibrium thermodynamics.

**Figure 1:**
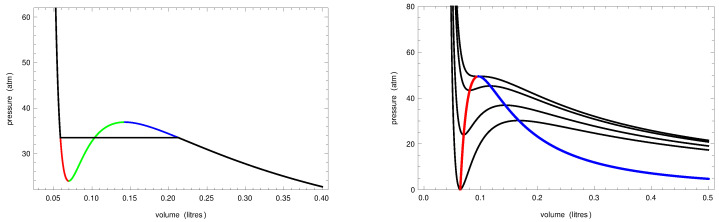
**Left**: the Maxwell equal area rule is used to replace part of the van der Waals isotherm with a horizontal segment yielding the isotherm describing the liquid–vapor transition (black curve). The van der Waals isotherm $\left(P+a/V^{2}\right)\left(V-b\right)=RT$, was plotted for $a=1.36\;\ell^{2}$ atm mol${}^{-2}$ and b=0.0319ℓ mol${}^{-1}$, at $T=140^{\circ }$ K, with R=0.082058ℓ atm mol${}^{-1}$. The equal area rule yields 33.48 atm as the value of the pressure at which vapor and liquid coexist. **Right**: black lines, from the bottom to the top, are van der Waals isotherms for the same *a* and *b* at temperatures $T=130,140,150,153.94^{\circ }$ K. The red and the blue curves are, respectively, the liquid and the vapor branches of the spinodal curve, which meet at the critical point $V_{\mathrm{crit}}=3b=0.0957\;\ell$ and $P_{\mathrm{crit}}=a/\left(27b^{2}\right)=49.50$ atm on the critical isotherm at $T_{\mathrm{crit}}=8a/\left(27Rb\right)=153.94^{\circ }$ K. Note that the values of *a* and *b* that we used are the ones valid for the oxygen, the experimental value of the critical temperature is $156^{\circ }$ K and the vapor pressure at temperature $140^{\circ }$ K is 27.50 atm [6].

The first attempts to provide a theoretical explanation of metastability are based on the real gas van der Waals equation [3], which, coupled with the Maxwell construction [4,5], can be interpreted as an equation describing the liquid–vapor transition (see the left panel of Figure 1). Below the critical temperature, the van der Waals isotherms have a non-monotonic behavior, but, using the *equal area* Maxwell rule, the kink can be replaced by a segment joining the high pressure part of the curve representing the liquid and the low pressure part of the curve representing the vapor.

At the points in the part of the original isotherm curve between the minimum and the maximum, called the *instability* branch of the isotherm, the compressibility is negative, since ∂v/∂p>0 (see the green part of the isotherm in the left panel of Figure 1). The locus of the minimum and maximum points of the sub-critical isotherms in the plane *V*–*P* is called the *spinodal curve*: the minimum and the maximum points form, respectively, the liquid and the vapor branches of the curve (red and blue curves in the right panel of Figure 1). All the points below the spinodal curve cannot represent equilibrium states of the real gas since they follow on the instability branch of one of the van der Waals isotherms and, thus, are mechanically unstable since they would have negative compressibility. Indeed, if a real gas is prepared in one of those states, the liquid and the vapor phase quickly separate through a mechanism called *spinodal* decomposition. On the other hand, if the real gas is prepared in states above the spinodal curve not belonging to the pure phase branches of the Maxwell isotherms, under particular experimental conditions, it is possible to observe metastable states, such as super-saturated vapor and super-heated liquid. It is thus rather natural to interpret those points (respectively, the red and the blue arcs in the left panel of Figure 1) of the van der Walls isotherms as metastable states.

Although several important studies tried to develop rigorous theories of metastability in the framework of thermodynamics and statistical mechanics of Gibbsian ensembles [7], it was soon clear that metastability is a genuine dynamical phenomenon that needs to be described by means of non-equilibrium statistical mechanics ideas. The first kinetical approach to metastability is the Becker–Doring theory [8], which dates back to 1935. However, almost half a century was needed to arrive to the first rigorous mathematical discussion of metastable states in the case of a simple mean-field spin system [9], i.e., the Curie–Weiss model. The necessity of a dynamical approach was also pointed out by many numerical studies; we refer to the review [10] and to the references therein.

In [9], the question of metastable states is posed in the framework of a stochastic spin system whose evolution is a Markov chain defined as a reversible Glauber dynamics. An external field is introduced in the Hamiltonian to select the *stable* homogeneous phase (corresponding to the minimum of the Hamiltonian). As an initial condition of the dynamics, the opposite state is considered, and its metastable character is shown by proving that the time needed by the system to hit for the first time the stable state is exponentially large with respect to the inverse temperature parameter. The method proposed in [9] is today known as the *pathwise approach* and has been used to study metastablity in a large variety of models. The first paper in which it was applied with success to a model with a physically acceptable short range interaction is [11], in which the existence of metastable states was proven for the Ising model.

The basic idea of the pathwise approach is searching for the optimal path in the configuration space connecting the metastable state to the stable one and computing its energy height. The time needed by the system to perform the transition from the metastable to the stable state, i.e., the *exit time*, is a random variable whose mean value can be estimated by an exponential function of the energy barrier that the system must overcome during the transition. The method provides also the portion of the configuration space explored by the system before performing the transition to the stable state and the tube of trajectories, called the *exit path*, followed during the exit excursion. Along the exit path, the system necessarily visits those particular configurations, called the *critical configurations* at which the optimal path attains its maximum. In the original version of the theory this was achieved via a detailed study of the configuration space and a suitable definition of *basin of attraction* of the metastable state based on the notion of cycles. The pathwise approach was further developed in [12,13,14]; see also [15]. Several techniques were introduced to simplify the application of the method. It is worth mentioning that, independently, a similar cycle theory was derived in [16,17] and applied to reversible Metropolis dynamics and to simulated annealing [18,19].

The general properties of the pathwise approach were further analyzed in [20,21,22,23,24] to disentangle the study of the transition time from that of the typical trajectories and to treat the irreversible system. This method was used to study the metastable behavior of the Ising model with isotropic and anisotropic interaction, in different dimensions, with different external magnetic fields evolving according to Glauber dynamics in [11,15,25,26,27,28,29,30]. Moreover, it has been used also for a variety of other models evolving according to Glauber dynamics, such as the Blume–Capel model in [21,31], the Potts model in [32,33], and hard-core model in [34,35,36]. Other applications of the pathwise approach are presented in [37,38,39,40] for the Metropolis dynamics and in [41,42,43] for parallel dynamics.

The focus of this paper is on the comparison between the metastable behavior of *serial* or *asynchronous* dynamics, i.e., stochastic spin systems in which the configurations are updated a site at a time, and *parallel* or *synchronous* dynamics, i.e., stochastic spin systems in which at each time step all the spins are simultaneously updated. Although the general ideas are similar, the parallel case is utterly more puzzling due to the very intricate structure of the possible paths that can be followed in the configuration space by the system during its random motion. In Section 2, we consider the Hamiltonian of the 2D Ising model and we show how it is possible to construct different dynamics by allowing or forbidding simultaneous spin updating. The structure of their critical configurations and optimal paths are discussed in Section 3.

Before closing the Introduction, it is worth mentioning two more approaches to the rigorous mathematical description of metastability which have been developed in the last decades. One is known as the *potential theoretic approach* and is based on the seminal papers [44,45]. We refer to [46] for an extensive discussion of this method and of its applications to several models. In the potential theoretic approach, the estimate of the hitting time is achieved through the use of the Dirichlet form and the spectral properties of the transition matrix. One of the advantages of this method is that it provides an estimate of the expected value of the transition time, including the prefactor, by exploiting a detailed knowledge of the critical configurations; see [46,47]. This method was applied in [48,49,50,51,52,53] to Metropolis dynamics and in [54] to parallel dynamics.

Finally, we mention the more recent *trace method*, firstly introduced in [55], that extends, in some sense, the pathwise approach and the potential theoretic approach. Differently from the pathwise approach, it does not rely on large deviations estimates, so it can be used to study models where the ratio between the jump rates is not exponential in the scaling parameter (e.g., condensing zero-range processes). Moreover, differently from the potential approach, the method does not depend on a reversibility assumption. The main idea is to consider a reduction in the process by removing rapid fluctuations from the trajectory. The authors considered indeed the trace of the process on the metastable states. Then, the metastability behavior is examined through a martingale problem that controls the convergence of the trace process. Moreover, based on the martingale characterization of Markov processes, sufficient conditions for metastability can be given (see [56] for a general review on the method and its applications).

## 2. Models

Let Λ be a finite square of $Z^{2}$ with periodic boundary conditions, namely, a two-dimensional finite torus. With each site i∈Λ, it is associated a *spin variable* σ(i)∈{−1,+1}. We denote by $\Omega ={\left\{-1,+1\right\}}^{\Lambda }$ the *configuration* or *state* space and we call *configuration* or *state* any element σ∈Ω. We say that two sites are *nearest neighbors* if, and only if, their Euclidean distance is equal to one. Given i∈Λ, we consider the *shift operator* $\Theta_{i}:\Omega \to \Omega$ which shifts a configuration so that the site *i* is mapped to the origin 0, that is to say ${\left(\Theta_{i}\left(\sigma \right)\right)}_{j}=\sigma_{i+j}$ for any j∈Λ. Given Δ⊂Λ, we denote by $\sigma_{\Delta }$ the restriction to Δ of a configuration σ∈Ω. Given σ∈Ω, we denote by $\sigma^{s}$, for s∈{−1,+1}, the configuration obtained by setting to *s* the value of the spin at the origin, namely, $\sigma^{s}\left(0\right)=s$ and $\sigma^{s}\left(i\right)=\sigma \left(i\right)$ for any i∈Λ\{0}.

A *pairwise interaction* is a collection of real numbers $J_{ij}$, for any i,j∈Λ, for i≠j, symmetrical and translationally invariant, which means that $J_{ij}=J_{ji}$ and $J_{ij}=J_{i+k,j+k}$ for all $k\in Z^{2}$. We shall also assume that the interaction is *finite range*, i.e., there exists I⊂Λ\{0} (not depending on the size of Λ) such that $J_{0i}\neq 0$ for any i∈I and $J_{0i}=0$ otherwise: it is worth noting that the symmetry of the interaction implies that *I* is symmetric with respect to the origin. We thus define the Hamiltonian

(1) H(σ)=−12∑i,j∈Λ:i≠jJijσ(i)σ(j)−h∑i∈Λσ(i),

with h∈R the *external magnetic field*, and consider on the single spin space the probability distribution

(2) $$f_{T,\sigma }\left(s\right)=\frac{\exp\{-H\left(\sigma^{s}\right)/T\}}{\exp\left\{-H\left(\sigma^{s}\right)/T\right\}+\exp\left\{-H\left(\sigma^{-s}\right)/T\right\}}$$

for any σ∈Ω. Note that the probability distribution $f_{T,\sigma }$ can be rewritten as

(3) $$f_{T,\sigma }\left(s\right)=\frac{1}{1+\exp\{\left[H\left(\sigma^{s}\right)-H\left(\sigma^{-s}\right)\right]/T\}}=\frac{1}{1+\exp\{-2s\left(\sum_{i\in I}J_{0i}\sigma \left(i\right)+h\right)/T\}},$$

which then implies

(4) $$f_{T,\sigma }\left(s\right)=\frac{1}{2}\left\{1+s\tanh h\left[\frac{1}{T}\left(\sum_{i\in I}J_{0i}\sigma \left(i\right)+h\right)\right]\right\}.$$

We shall consider stochastic evolutions in Ω modeled as Markov chains in which single spins are updated according to the probability distribution (2). The dynamics can be implemented by allowing one spin change at a time or the simultaneous change of all the spins on the lattice, the dynamics will be respectively called *asynchronous* or *synchronous*.

### 2.1. Asynchronous Dynamics

The *heat bath* dynamics is defined as the discrete time Markov chain $\sigma_{t}\in \Omega$, with $t\in Z_{+}$, with updating the rule defined as follows: at time *t* a site *i* is chosen at random with uniform probability 1/|Λ| and the configuration $\sigma_{t}$ is constructed by letting $\sigma_{t}\left(j\right)=\sigma_{t-1}\left(j\right)$ for any j≠i and $\sigma_{t}\left(i\right)=s$ with probability $f_{T,\Theta_{i}\sigma_{t-1}}\left(s\right)$. In other words, the heat bath dynamics is the discrete time Markov chain with transition probability defined as follows: for σ,η∈Ω such that σ≠η

(5) $$p_{T}\left(\sigma ,\eta \right)=\left\{\begin{array}{cc} \frac{1}{\left|\Lambda \right|}f_{T,\Theta_{i}\sigma }\left(\eta \left(i\right)\right)\; & \mathrm{if}\;\exists i\in \Lambda :\eta (i)\neq \sigma (i)\;\mathrm{and}\;\eta (j)=\sigma (j)\;\mathrm{for}\;j\neq i\\ 0\; & \mathrm{otherwise} \end{array}\right.$$

and

(6) $$p_{T}\left(\sigma ,\sigma \right)=1-\sum_{\eta \in \Omega \backslash \{\sigma \}}p_{T}\left(\sigma ,\eta \right).$$

As it is well known (Section 4.5.1 in [57]), the heat bath dynamics is *reversible* with respect to the *Gibbs measure* on Ω

(7) $$\mu_{T}\left(\sigma \right)=\frac{1}{Z_{T}}e^{-H(\sigma )/T}$$

where the *partition function* is

(8) $$Z_{T}=\sum_{\sigma \in \Omega }e^{-H(\sigma )/T},$$

that is to say, the *detailed balance condition*

(9) $$\mu_{T}\left(\sigma \right)p_{T}\left(\sigma ,\eta \right)=\mu_{T}\left(\eta \right)p_{T}\left(\eta ,\sigma \right)$$

is satisfied for any σ,η∈Ω. This, together with the fact that the Markov chain is irreducible and the state space is finite, implies that the stationary measure is unique and it is given by the Gibbs measure.

Asynchronous models are typically used in statistical mechanics to introduce stochastic versions of statistical spin systems in order to study how equilibrium is approached. Often, for efficiency reasons, the Metropolis algorithm (Section 3.1 in [57]) is preferred to heat bath and to other similar rules.

A crucial role in the study of the metastable behavior of the dynamics is played by the so-called energy cost. Consider two configurations, σ and η, differing by only the spin at site *i*; we have that, in the limit T→0, p(σ,η)≈1/|Λ| if H(η)<H(σ) and p(σ,η)≈exp{−[H(η)−H(σ)]/T}/|Λ| if H(η)>H(σ). Then, it is reasonable to define the *energy cost* of the transition from σ to η as the quantity Δ(σ,η)=0 if H(η)<H(σ) and Δ(σ,η)=H(η)−H(σ) if H(η)>H(σ).

### 2.2. Synchronous Dynamics

A model in which all the spins are updated at each time independently and simultaneously with the probability distribution (2) can be defined as the Markov chain $\sigma_{t}\in \Omega$, with $t\in Z_{+}$, with transition matrix

(10) $$p_{T}\left(\sigma ,\eta \right)=\prod_{i\in \Lambda }f_{T,\Theta_{i}\sigma }\left(\eta \left(i\right)\right).$$

This model is an example of *reversible probabilistic cellular automata* (PCA); see, for example, [58,59,60]. We mention that the class of reversible PCA is slightly larger, indeed, the constraint that the set *I* in (4) does not contain the origin can be relaxed; when the origin is considered in *I*, as, for instance, in [41,61], its contribution is called the *self-interaction* term. It does not appear in our derivation of reversible PCA, since we started from the Hamiltonian (1), where it would appear simply as a constant additive irrelevant contribution (Section 4.5.1, Equation (4.38) in [57]).

As it is proven in [62,63], reversible probabilistic cellular automata satisfy the detailed balance condition

(11) $$\mu_{T}\left(\sigma \right)p_{T}\left(\sigma ,\eta \right)=\mu_{T}\left(\eta \right)p_{T}\left(\eta ,\sigma \right)$$

with respect to the Gibbs measure on Ω

(12) $$\mu_{T}\left(\sigma \right)=\frac{1}{Z_{T}}e^{-G_{T}\left(\sigma \right)/T}$$

where

(13) $$Z_{T}=\sum_{\sigma \in \Omega }e^{-G_{T}\left(\sigma \right)/T}$$

is the partition function and

(14) $$G_{T}\left(\sigma \right)=-h\sum_{i\in \Lambda }\sigma \left(i\right)-T\sum_{i\in \Lambda }\log\cosh\left[\frac{1}{T}\left(\sum_{j\in i+I}J_{ij}\sigma \left(j\right)+h\right)\right]$$

for any σ∈Ω. Thus, even in the synchronous case, we have that the stationary measure is unique, but it is different from the Gibbs measure (7) found for the statistical mechanics model considered in the asynchronous case.

It is interesting to note that the Gibbs measure (12) is such that $G_{T}=-T\log\left(Z_{T}\mu_{T}\right)$ depends on the temperature, which is not the case in standard statistical mechanics models. On the other hand, the global minima of the function

(15) $$H\left(\sigma \right)=\lim_{T\to 0}G_{T}\left(\sigma \right)=-h\sum_{i\in \Lambda }\sigma \left(i\right)-\sum_{i\in \Lambda }|\sum_{j\in i+I}J_{ij}\sigma \left(j\right)+h|$$

are the configurations in which the dynamics will be trapped at a low temperature. The low temperature regime is indeed the limit of interest in the study of metastablity. By abusing the notation, the function *H* is called *Hamiltonian* or *energy*, and its global minima are called *ground states*. Moreover, a crucial role in our discussion is played by the *cost function*

(16) Δ(σ,η)=−limT→0TlogπT(σ,η)=∑i∈Λ:η(i)[∑j∈i+IJijσ(j)+h]<02|∑j∈i+IJijσ(j)+h|,

since, as it was proven in (Section 2.6 in [41]), for the transition matrix, it is possible to prove the so-called Friedlin–Wentzel condition

(17) $$e^{-\Delta (\sigma ,\eta )/T-\gamma (T)/T}\leq \pi_{T}\left(\sigma ,\eta \right)\leq e^{-\Delta (\sigma ,\eta )/T+\gamma (T)/T}$$

with γ(T)→0 as T→0. Equation (17), together with the equality

(18) H(σ)+Δ(σ,η)=H(η)+Δ(η,σ),

which follows immediately from (11), (12), (15) and (16), allows us to interpret Δ(σ,η) as the energy cost that the chain has to pay in the jump from σ to η. We note that it is possible that both Δ(σ,η) and Δ(η,σ) are positive. This is not the case in the asynchronous case, in which only one of the two can be positive.

### 2.3. The Ising Case

In this paper, we focus our discussion on the Ising case, namely, we assume that the interaction is 1 for nearest neighbors spins and 0 otherwise, which mean that the set *I* is equal to set of the four neighboring sites of the origin. With this choice, the Hamiltonian (1) of the asynchronous model is the standard 2D Ising Hamiltonian

(19) H(σ)=−12∑i,j∈Λ:|i−j|=1σ(i)σ(j)−h∑i∈Λσ(i).

On the other hand, the energy of the synchronous version of the dynamics reads

(20) H(σ)=−h∑i∈Λσ(i)−∑i∈Λ|∑j∈Λ:|j−i|=1σ(j)+h|.

**Figure 2:**
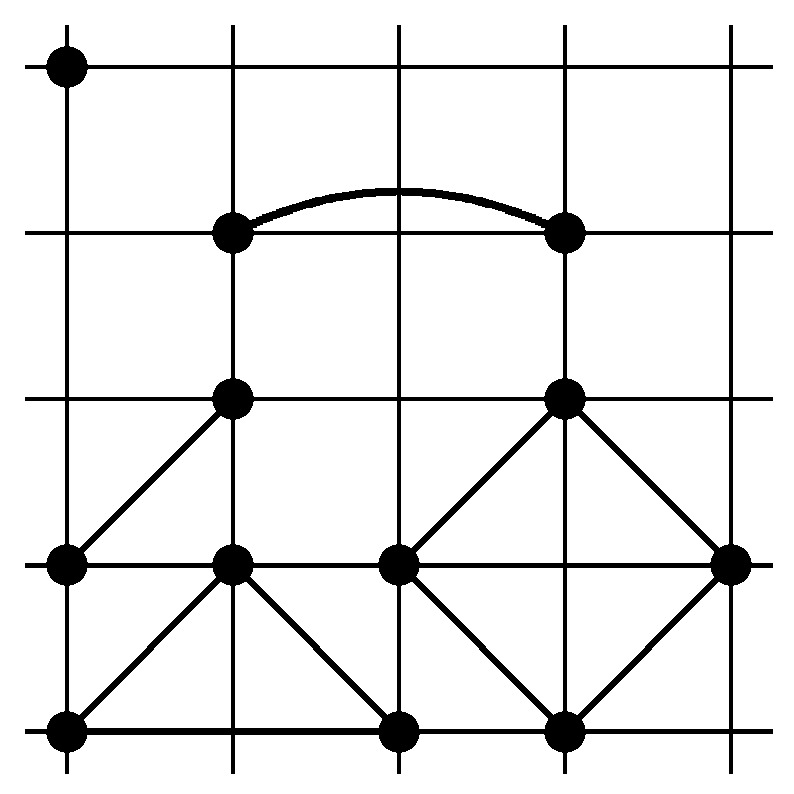
Schematic representation of coupling constants (22).

In statistical mechanics models, Hamiltonians are usually written in terms of coupling constants. Following [64], see also [65,66], for the Hamiltonian (20) we obtain

(21) $$\begin{array}{cc} H(\sigma )=\; & \;\;\;-J_{.}\sum_{x\in \Lambda }\sigma \left(x\right)-J_{{}_{\langle \langle \rangle \rangle }}\sum_{\langle \langle xy\rangle \rangle }\sigma \left(x\right)\sigma \left(y\right)-J_{{}_{\langle \langle \langle \rangle \rangle \rangle }}\sum_{\langle \langle \langle xy\rangle \rangle \rangle }\sigma \left(x\right)\sigma \left(y\right)\\ \; & \;\;\;-J_{{}_{▵}}\sum_{{▵}_{xyz}}\sigma \left(x\right)\sigma \left(y\right)\sigma \left(z\right)-J_{{}_{◊}}\sum_{{◊}_{xywz}}\sigma \left(x\right)\sigma \left(y\right)\sigma \left(w\right)\sigma \left(z\right) \end{array}$$

where the coupling constants

(22) $$J_{.}=\frac{5}{2}h,\;J_{{}_{\langle \langle \rangle \rangle }}=1-\frac{1}{4}h,\;J_{{}_{\langle \langle \langle \rangle \rangle \rangle }}=\frac{1}{2}-\frac{1}{8}h,\;J_{{}_{▵}}=-\frac{1}{8}h,\;J_{{}_{◊}}=-\frac{1}{2}+\frac{3}{8}h,\;$$

refer, respectively, to single site, next to the nearest neighbor pairs, third neighbor pairs, triangle, and diamond clusters (see, Figure 2). Note that the diamond cluster coupling is negative for small magnetic field, which yields an anti-ferromagnetic behavior of the interaction that will give rise to very peculiar phenomena that are discussed in the following sections. The triangle cluster coupling is negative, as well, but it becomes negligible for small *h*.

We stress that implementing the dynamics in asynchronous or synchronous ways yields completely different stochastic models. We have already noticed that the Hamiltonians are different, and the simple 2D Ising Hamiltonian has to be compared to the complicated Hamiltonian (21) found in the synchronous case. Even the cost functions are fairly different: in the asynchronous case, it reduces to the positive part of the energy difference between configurations differing for the value of one spin, whereas in the synchronous case, it is defined for any pair of configurations and it is given by the baroque formula (16). However, we want to remark that in the latter case, the energy cost is defined for any pair of configurations because in a single step, the system can jump from any configuration to any other. This is the essential difficulty of studying metastability for synchronous dynamics: the fact that the structure of the trajectories followed by the system in the configuration space is utterly complicated due to the fact that any transition is possible in a single time step.

## 3. Metastable State and Critical Configurations

As already mentioned in Section 1, the first rigorous and full description of metastable behavior from the mathematical point of view dates back to paper [9], where the theory of the pathwise approach was firstly introduced. Other widely applied methods are nowadays known as the potential theoretic approach and the trace method, respectively proposed in [44,45,55]. For a comprehensive discussion of the pathwise and the potential theoretic approach, we refer the reader to the books [15,46].

Here, we shall review some metastability results adopting the pathwise point of view; in particular, we shall follow the strategy refined in [20] for the synchronous dynamics with the Metropolis updating algorithm and extended in [22] to a much larger class of Markov chains, including not reversible dynamics. This theory applies both to the heat bath Glauber dynamics and to the reversible PCA. We warn the reader that we use the same notation (*H* for Hamiltonian, Δ for the energy cost, etc.) for the two cases, but, depending on the model one has in mind, the correct related quantities should be used.

### 3.1. General Results

A *path of length* n≥1 is a sequence $\left\{\omega_{1},\ldots \omega_{n}\right\}\in \Omega^{n}$ such that $p_{T}\left(\omega_{i},\omega_{i+1}\right)>0$ for any i=1,…,n−1. We denote by F the set of all loop free paths and, given that $A,A^{\prime }\subset \Omega$ is not empty, we let $F(A,A^{\prime })$ be the set of all loop free paths with the first configuration in *A* and last configuration in $A^{\prime }$. Given a path ω of length *n*, we define the *height* of the path as

(23) $$\Phi_{\omega }=\max_{i=1,\ldots ,n-1}\left[H\left(\omega_{i}\right)+\Delta \left(\omega_{i},\omega_{i+1}\right)\right].$$

Given $A,A^{\prime }\subset \Omega$, the *communication height* $\Phi (A,A^{\prime })$ between *A* and $A^{\prime }$ is defined as

(24) $$\Phi \left(A,A^{\prime }\right)=\min_{\omega \in F(A,A^{\prime })}\Phi_{\omega }.$$

We define the *stability level* $V_{\sigma }$ of any configuration σ∈Ω as the minimal height, with respect to H(σ), which must be overcome by paths connecting σ to the set of configurations at energy smaller than H(σ), namely,

(25) $$V_{\sigma }=\Phi \left(\left\{\sigma \right\},\left\{\eta \in \Omega :\;H\left(\eta \right)<H\left(\sigma \right)\right\}\right)-H\left(\sigma \right).$$

We are now ready to give the key notion of the metastable state. We let $\Omega^{s}$ be the set of the absolute minima of the Hamiltonian, namely, the set of ground states, and define the *maximal stability level*

(26) $$\Gamma =\max_{\sigma \in \Omega \backslash \Omega^{s}}V_{\sigma }>0$$

and the set of *metastable* states

(27) $$\Omega^{m}=\left\{\sigma \in \Omega \backslash \Omega^{s}:\;V_{\sigma }=\Gamma \right\}.$$

The set $\Omega^{m}$ deserves the name of the set of metastable states since it is possible to prove the following theorem (Theorem 2.1 in [22]): for any $\sigma \in \Omega^{m}$, for any ε>0 we have that

(28) $$\lim_{T\to 0}P_{\sigma }\left(e^{(\Gamma -\varepsilon )/T}<\tau_{\Omega^{s}}<e^{(\Gamma +\varepsilon )/T}\right)=1,$$

where $P_{\sigma }$ is the probability for the chain $\sigma_{t}$ started at σ and the random variable $\tau_{\Omega^{s}}$ is the *first hitting time* to $\Omega^{s}$ for the dynamics started at σ, i.e., $\tau_{\Omega^{s}}=\inf\left\{t\geq 0:\;\sigma_{t}\in \Omega^{s}\right\}$. In words, Equation (28) states that the time needed by the system to exit the metastable state and touch the ground state is, controlled in probability, of order exp{Γ/T}. Thus, (28) gives a mathematically rigorous meaning to the statement “the exit time from the metastable state is of order exp{Γ/T}”.

The pathwise approach provides also an estimate for the mean value $E_{\sigma }\left[\tau_{\Omega^{s}}\right]$; indeed, it is possible to prove that

(29) $$\lim_{T\to 0}T\log E_{\sigma }\left[\tau_{\Omega^{s}}\right]=\Gamma ,$$

see (Theorem 2.2 in [22]).

The exit time is, for sure, the main property of metastable states that one wants to compute. Another relevant property concerns the path followed by the system to exit the metastable state. Think of a super-saturated vapor: how does it perform the transition to the liquid stable phase? Does it happen through the coalescence of small droplets of the liquid phase that appear throughout the whole volume occupied by the system? Or does it happen through a sudden formation of a sufficiently large droplet? These questions can be answered in the framework of the pathwise approach, indeed it is possible to identify configurations, called *critical*, that must be necessarily visited during the excursion from the metastable to the stable state. These special configurations will give a clear indication of the mechanism of the transition from the metastable to the stable state.

To make this formal, given that $A,A^{\prime }\subset \Omega$ is not empty, we define the set of *optimal paths* connecting *A* to $A^{\prime }$, and denote it by $F_{o}\left(A,A^{\prime }\right)$, as the set of loop free paths $\omega \in F(A,A^{\prime })$ such that $\Phi_{\omega }=\Phi \left(A,A^{\prime }\right)$. That is to say, an optimal path connecting *A* to $A^{\prime }$ is a path starting in *A*, ending in $A^{\prime }$, and having maximal height equal to the communication height between *A* and $A^{\prime }$.

**Figure 3:**
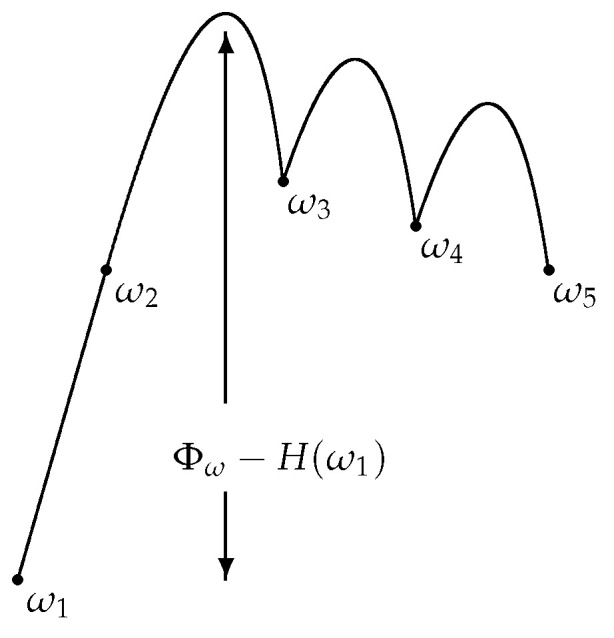
Schematic representation of the height of a path.

Morally, the critical configurations are those configurations, where the optimal paths attain the maximal height. Unfortunately, as remarked above and depicted in Figure 3, the maximal height of a path in general does not correspond to the energy of one of the configurations forming the path. This is true in the case of Glauber dynamics [20], but it is not necessarily true in a more general set-up, including the PCA case. Thus, a more sophisticated notion of critical configuration is needed [22]: given η∈Ω and $A^{\prime }\subset \Omega$, the set of *saddles* $S(\left\{\eta \right\},A^{\prime })$ between η and $A^{\prime }$ is the set of configurations ξ such that there exists an optimal path $\omega \in F_{o}\left(\left\{\eta \right\},A^{\prime }\right)$ and a configuration ζ such that ξ follows ζ in the path ω and $H\left(\zeta \right)+\Delta \left(\zeta ,\xi \right)=\Phi (\left\{\eta \right\},A^{\prime })$.

Among all the possible saddles, a relevant role is played by those that must be necessarily visited by optimal paths: given η∈Ω and $A^{\prime }\subset \Omega$ a subset $W\subset S(\left\{\eta \right\},A^{\prime })$ is a *gate* for η and $A^{\prime }$ if and only if every optimal path in $F_{o}\left(\left\{\eta \right\},A^{\prime }\right)$ intersects *W*. Moreover, a gate *W* is *minimal* if, and only if, for any $W^{\prime }\subset W$ and $W^{\prime }\neq W$ there exists an optimal path which does not intersect $W^{\prime }$. The fact that gates must be necessarily visited during the transition from the metastable to the stable state is proven in (Theorem 2.4 in [22]) stating that given $\sigma \in \Omega^{m}$ and *W*, a minimal gate for σ and $\Omega^{s}$, there exists c>0 such that

(30) $$P_{\sigma }\left[\tau_{W}>\tau_{\Omega^{s}}\right]\leq e^{-c/T},$$

where $\tau_{W}$ is the first hitting time to *W* for the dynamics started at σ.

We mention that in the framework of the pathwise approach, it is also possible to characterize the behavior of the system before it performs the transition to the metastable state. Indeed, by using the theory of cycles, it is possible to define the basin of attraction of the metastable state and to prove that in a time smaller than the exit time, the system is confined to move within such a basin of attraction.

Finally, we remark that the estimate provided by the pathwise approach for the exit time is rather rough, in the sense that it is given at the level of logarithmic equivalence. A more refined estimate can be provided in the framework of the potential theoretic approach, using the trace method. The typical result that can be proven is the following: for T→0,

(31) $$\frac{E_{T}\left[\tau_{\Omega^{s}}\right]}{\exp\{\Gamma /T\}}=C\left(\Lambda \right)\left[1+o\left(1\right)\right]$$

where C(Λ) is a constant, depending on the volume Λ, which can be computed in terms of the number of saddles between the starting metastable state σ and the set of ground states $\Omega^{s}$.

The results that we have discussed in this section are general, but the model dependent inputs that are presented in Section 3.2 and Section 3.3 are necessary if one wants to describe the metastable behavior of a specific system. This is precisely the idea on which the paper [20] is based and which was further developed in [22]: disentangling the proof of the general properties of metastable states from that of the model dependent inputs. These model-dependent inputs, necessary to achieve the full characterization of the metastable behavior of a particular model, are the set of metastable states and the optimal paths connecting such states to the set of stable ones. Once we obtain this, all the properties follow from the general theory.

### 3.2. Metastable Behavior of the 2D Ising Model

We consider the asynchronous dynamics (heat bath) introduced in Section 2.1 for the standard 2D Ising Hamiltonian considered in Section 2.3 with 0<h≪1 such that 2/h is not an integer. This assumption reduces the number of degenerate critical configurations; we refer to [20] for a thorough discussion of the case in which 2/h is an integer and to [11], page 213, where some comments on this singular point are reported. Although the metastable behavior for such a model was first studied in [11] in the case of the Metropolis single site updating rule, we describe here the main results using the language developed in the previous sections.

**Figure 4:**
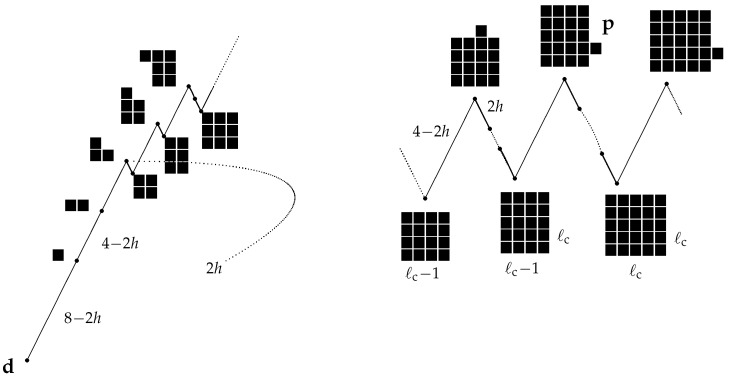
Schematic representation of the optimal path between d and u for the Ising model with indication of energy differences. Black squares represent pluses.

We denote by d and u the two homogeneous configurations with spins respectively equal to −1 and +1. It is not surprising that $\Omega^{s}=\left\{u\right\}$ and $\Omega^{m}=\left\{d\right\}$, provided the Hamiltonian is the standard 2D Ising Hamiltonian defined in (19). The optimal path connecting d to u is depicted in Figure 4: four minuses are flipped to plus one after the other to form a two by two square, the sides of the droplet grow one after the other in order to obtain alternately a rectangle of pluses with side length difference equal to one and a square of pluses till u is reached. A side is added to a rectangle (or a square) by flipping to plus a minus spin adjacent to the rectangle (adding one protuberance) and then flipping, one after the other, minus spins with two adjacent pluses until a rectangular or square shape is recovered.

When a plus protuberance is added to the side of the plus rectangle (or square) of length *ℓ*, the energy increases by 4−2h; on the other hand, when this very side is filled by pluses, the energy decreases by 2h(ℓ−1). The energy difference related to the process of adding a side is equal to 4−2h−2h(ℓ−1), which is positive provided $\ell <\ell_{c}$, where $\ell_{c}=\left\lfloor 2/h\right\rfloor +1$, where, for any real number *a*, ⌊a⌋ is its integer part, i.e., the largest integer smaller than *a*. Thus, the optimal path achieves its maximal energy at configuration p (see Figure 4) made of a $\ell_{c}\left(\ell_{c}-1\right)$ rectangle of pluses with a unit protuberance on one of its longest sides. The communication height between d and u is

(32) $$\Gamma =H\left(p\right)-H\left(d\right)=8\ell_{c}-2h\left[\ell_{c}\left(\ell_{c}-1\right)+1\right]∼\frac{8}{h},$$

where *H* is the Ising Hamiltonian (19) and the estimate is valid for h→0.

With the model-dependent components summarized in this section, the results reported in Section 3.1 provide a full description of the metastable behavior of the Ising model: the state d is metastable and, if the system is prepared in d, at small temperature, the typical time necessary to reach the stable state u is of order exp{8/(hT)}. Moreover, during the transition from the metastable to the stable state, with high probability, the system visits the configuration p. This last remark is very important since it means that the transition to the stable state is performed through the sudden nucleation of a large critical droplet and not via the coalescence of many small droplets distributed throughout the whole volume Λ.

### 3.3. Metastable Behavior of the Reversible Nearest—Neighbor PCA

We consider the synchronous dynamics (PCA) introduced in Section 2.2 for the standard 2D Ising Hamiltonian considered in Section 2.3 with 0<h≪1 such that 2/h is not an integer. We also assume that the side length of the lattice Λ is an even number. The metastable behavior for such a model was first studied in [67]; we describe here the main results using the language developed above.

As in the previous section, we denote by d and u the two homogeneous configurations with spins respectively equal to −1 and +1. Moreover, we consider the two chessboard configurations such that all the spins associated with sites on the even sub-lattice are equal and opposite to the spins associated with sites on the odd sub-lattice. These two identified configurations are denoted by c. The two chessboards are identified, since, with high probability at low temperature, the chain started at one of them is trapped in a continuous flip-flop among the two, which is performed with no energy cost. The presence of this flip-flopping configuration is a signature of the parallel (synchronous) character of the dynamics. It is proven in [67] that $\Omega^{s}=\left\{u\right\}$ and $\Omega^{m}=\left\{d,c\right\}$, with respect to the Hamiltonian (20). The fact that chessboard configurations play a crucial role in the metastable behavior of the model is also due to the antiferromagnetic term $J_{⋄}$, which is present in the Hamiltonian (21).

**Figure 5:**
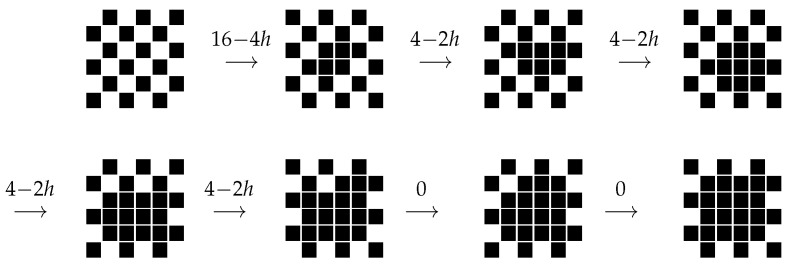
Schematic representation of the optimal path between c and u for the PCA model with indication of the energy cost computed using (16) with *I*, the set of nearest neighbors and $J_{ij}=1$ for *i* and *j* nearest neighbors. Black squares represent pluses, white ones minuses.

The optimal path connecting c to u is constructed—see also (Section 5.2, case A${}_{3}$–A${}_{4}$ in [43])—by letting the spins perform a flip-flop at each time, except for some pluses that are kept fixed in such a way to eventually invade the whole lattice (see Figure 5): at the first step, two arbitrary next-to-the-nearest pluses (Euclidean distance $\sqrt{2}$) are kept fixed so that a two by two plus square is formed in the chessboard sea. At the second step, the pluses in the square and a plus adjacent to the square are kept fixed in order to form a two by three rectangle. At the third step, the pluses in the rectangle are kept fixed together with the plus at the center of one chessboard side adjacent to the longest side of the rectangle. In this way, a three by three plus rectangle is formed. Before taking the fourth step, the chessboard sea is allowed to flip-flop, if necessary, so that a plus appears at the site in the center of one of the chessboard sides adjacent to the plus square, thus such a spin is kept fixed and a three times four rectangle is formed. In the fifth step, one of the pluses adjacent to the longest side of the plus rectangle is kept fixed, and a double or triple plus protuberance is formed on such a side. In the following steps, this protuberance is kept fixed together with the plus in the three by four rectangle till the four by four plus rectangle is formed. This path is then followed, alternating plus squares and plus rectangles alike, whose side lengths differ by one.

When one plus adjacent from the exterior to a square or a rectangle of pluses is kept during the flip-flop, the energy cost is equal to 4−2h. This is the total cost paid to add a plus slice, since the following steps have no cost. On the way back, eroding a plus slice of length *ℓ* has a cost of 2h(ℓ−1) since at each step, the external pluses, having only two neighboring pluses, are flipped, paying the cost 2h The last step is cost free because the last plus has just one neighboring plus. By repeating the computation performed in the asynchronous case, one finds again the critical length $\ell_{c}=\left\lfloor 2/h\right\rfloor +1$ for the plus droplets in the chessboard sea. Thus, the optimal path achieves its maximal height in the jump from the $\ell_{c}\times \left(\ell_{c}-1\right)$ plus rectangle to the configuration in which a double or triple plus protuberance is added to the slab adjacent to the longest side of the same rectangle. Finally, the communication height between c and u is

(33) $$\Gamma =H\left(q_{2}\right)-H\left(c\right)+2h\left(\ell_{c}-1\right)=-2h\ell_{c}^{2}+\left(8+2h\right)\ell_{c}+4h∼\frac{8}{h},$$

where $q_{2}$ is the $\ell_{c}\times \ell_{c}$ plus droplet in the sea of the chessboard, *H* is the Hamiltonian (20) and the estimate is valid for h→0.

We do not describe in detail the optimal path between d and u. We just mention that it is made of two parts: the first part from d to c realizes the growth of the chessboard square droplet $q_{1}$ of size ⌊2/h⌋+1 in the sea of minuses and its eventual growth to c, (see (Section 5.2, case A${}_{1}$–A${}_{2}$ in [43])); the second part is precisely the optimal path from c to u, (see also (Section 5.2, case A${}_{3}$–A${}_{4}$ in [43])). Its maximal height is equal to the value Γ computed in (33), and this explains why this model has two metastable states.

With the model-dependent components summarized in this section, the results reported in Section 3.1 provide a full description of the metastable behavior of the Ising model: the states d and c are metastable and, if the system is prepared in d, at small temperature, the typical time necessary to reach the stable state u is of order exp{8/(hT)}. Moreover, during the transition from the metastable to the stable state, with high probability, the system visits the configurations $q_{1}$, c, and $q_{2}$.

This situation has been called in the literature a “series of metastable states”. We refer the interested reader to the papers [51] for some general results and their applications to the case of the Blume–Capel model with zero chemical potential [68] for the discussion of this phenomenon in the context of probabilistic cellular automata, and to [43] for the extension of these results to an arbitrary series of metastable states. The case of the Blume–Capel model is also discussed in [69].

### 3.4. Numerical Simulations

We illustrate by means of numerical simulations the nucleation phenomenon described on a rigorous basis above. We refer to [70] for a detailed numerical study of the exit time for the 2D Ising model.

**Figure 6:**
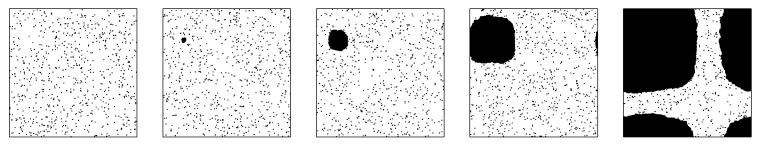
Nucleation of the plus phase in the asynchronous Ising model with h=0.2 and 1/T=0.78. From the left to the right, the configurations after 800, 1000, 1400, 2000, and 3200 Monte Carlo full lattice sweeps of the lattice are reported. Black and white spots correspond to plus and minus spins, respectively.

We first consider the asynchronous model, and we simulate the stochastic Ising model on the 512×512 lattice with magnetic field h=0.2. In Figure 6, we show the nucleation of the plus phase starting from the minus metastable state at inverse temperature 1/T=0.78. The sequence of configurations shows that the nucleation is performed via the formation of a single droplet of pluses in the sea of minuses. Due to the fact that in the simulation we could not consider a too small value of *T*, the droplet is not perfectly rectangular, and the sea of minuses is full of very small sub-critical droplets of pluses.

**Figure 7:**
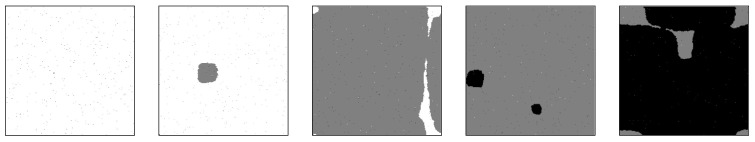
Nucleation of the plus phase in the synchronous nearest neighbor PCA model with h=0.3 and 1/T=0.90. From the left to the right, the configurations after 3000, 3600, 6000, 7600, and 9600 Monte Carlo full lattice sweeps of the lattice are reported. Configurations on 2×2 tiles are reported using grayscale from −4 (white) to +4 (black).

In Figure 7, we illustrate the more complex nucleation phenomenon for the asynchronous nearest neighbor PCA model on the 512×512 lattice with magnetic field h=0.3 and 1/T=0.90. In order to show the nucleation of the chessboard configuration, we represent the whole configuration on the 256×256 lattice, and we associate with each site of the new lattice the sum of the spins in a 2×2 tile of the original lattice. The new block variables, taking values in {−4,−2,0,+2,+4}, are plotted using a grayscale paddle ranging from white (−4) to black (+4). The sequence of configurations shows that the nucleation of the chessboard (gray) configuration is performed via the formation of a single droplet in the sea of minuses. Then, the plus phase is nucleated in the sea of chessboard via the formation of a droplet of pluses. Due to the relatively high value of the temperature that we used in the simulations, the formation of two plus droplets is observed.

**Figure 8:**
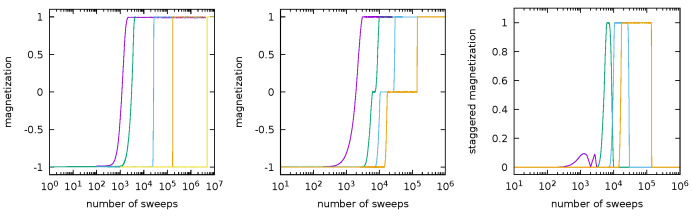
Magnetization and staggered magnetization versus the number of full Monte Carlo lattice sweeps. **Left**: magnetization of the asynchronous Ising model on the 512×512 lattice with h=0.2 and 1/T=0.70,0.79,0.80,0.85,0.90 (respectively from the left to the right, i.e., purple, green, blue, orange, and yellow). **Center**: magnetizations of the synchronous nearest neighbor reversible PCA on the 512×512 lattice with h=0.3 and 1/T=0.85,0.90,0.95,1.00 (respectively from the left to the right, i.e., purple, green, blue, and orange). **Right**: staggered magnetization for the same model as in the center panel.

In order to give a vivid idea of the time scales involved in the nucleation process, we plot in Figure 8 the magnetization, namely, the sum of all the spins on the lattice, versus the number of full Monte Carlo sweeps for the synchronous and asynchronous dynamics started at the minus configuration. A full Monte Carlo sweep corresponds to one step of the dynamics in the synchronous case and to a sequence of steps equal to the number of sites of the lattice in the asynchronous case.

It is not reasonable to compare the time scales observed in the figure to the result (29), which is an exact prediction for the exit time in the limit T→0. Indeed, in simulating metastability effects, it is necessary to use not very small values of the temperature; otherwise, the dynamics would stick to the metastable state and nothing would be observed. We refer to (Figure 3 in [37]) for the numerical estimate of the exit time for the Ising model with free boundary conditions on a small lattice.

Nevertheless, it is very interesting to remark how neatly the simulations show, in both cases, that the exit time from the metastable state increases dramatically when *T* becomes smaller and smaller (note that in the figure, the horizontal scale is logarithmic). Moreover, the data in the pictures also show that the exit from the metastable state is an abrupt phenomenon: the system is trapped in the metastable state for a long time, but when it performs the transition, this process is completed in a very small time.

It is also very interesting to observe that, in the PCA case, the dynamics started at the minus configuration, before reaching the plus state, spends a huge time in a configuration with zero magnetization, namely, the chessboard metastable state. This effect is lost if the temperature is so large (purple curve) that fluctuations dominate with respect to the behavior driven by the energy landscape. The fact that the intermediate zero magnetization state is precisely the flip-flopping chessboard configuration is demonstrated by the right panel, where we plotted the staggered magnetization, i.e., the absolute value of the difference between the sum of the spins on the even sub-lattice and the sum of the spin on the odd one, versus the number of full sweeps.

## 4. Concluding Remarks

We showed that starting from the same spin system Hamiltonian (1), depending on how the spin changes are implemented, asynchronously or synchronously, two different stochastic models are found. The two models differ not only for their stationary behavior, i.e., the stationary Gibbs measure, but also for their dynamical behavior. In particular, we discussed these dynamical aspects in the framework of the metastability theory and, in this connection, we showed that, depending on how the dynamics is implemented, different metastable states can be found. Moreover, the critical configurations and the exit time also turn out to be different.

In this review, we compared the asynchronous heat bath dynamics for the Ising model with its natural parallel implementation. However, one could have considered the parallel implementation of asynchronous Metropolis dynamics, as done in [71]. Here, indeed, a 2D Ising model with Hamiltonian (19) is considered, and the spins on the even (odd) sub-lattice are simultaneously updated by using the Metropolis weights [38]. Given a random set of sites *I* belonging to the even (odd) sub-lattice, and sampled under ν, the transition matrix is given by

(34) $$p_{T}\left(\sigma ,\eta \right)=\left\{\begin{array}{cc} \;\;\frac{\nu (I)}{\left|\Lambda \right|}e^{-{\left[H\left(\eta \right)-H\left(\sigma \right)\right]}_{+}/T}\; & \;\;\mathrm{if}\;\eta (j)=-\sigma (j)\;\mathrm{for}\;j\in I,\eta (j)=\sigma (j)\;\mathrm{for}\;j\in \Lambda \backslash I,\\ \;\;0\; & \;\;\mathrm{otherwise} \end{array}\right.,$$

where, for any a∈R, the positive part ${\left[a\right]}_{+}$ of *a* is equal to *a* if a>0 and to 0 otherwise. The Markov chain defined by (34) is reversible with respect to the Gibbs measure defined in (7). Despite the fact that new jumps are allowed by the dynamics, in [71], it was shown that the metastable character of the one-site Metropolis dynamics is preserved. The key observation is that the flip of the spins in *I* can be substituted by a suitable sequence of sequential single spin flip events. Since the sites in *I* belong to the same sub-lattice, the author could prove that a configuration is a local minimum for the multi-spin dynamics if, and only if, it is a local minimum in the single spin flip case. A natural generalization of the model might be to consider a general set *I*, not necessarily belonging to a sub-lattice. In this case, the set *I* might be decomposed in a connected (in the sense of nearest neighbors) part and in a non-connected part. The non-connected part might behave as the case in [71], while the connected cluster poses extra challenges and might change drastically the metastability scenarios.

As we mentioned in Section 2.2, in this review, we did not consider the case of PCA with non-null *self-interaction*, that is to say, the case in which $J_{00}\neq 0$ in Equation (3) (i.e., the set *I* contains the origin) since its dynamics cannot be written in terms of heat bath rates. However, for $J_{00}\neq 0$ interesting and different metastability scenarios arise: the case $J_{00}=1$ was studied in [41] showing a similar metastable behavior to the Ising model with the asynchronous dynamics. For $0<J_{00}<1$, Ref. [42] showed that, quite surprisingly, results similar to those found in [31] for the Blume–Capel model were obtained.

As we stressed in our manuscript, the synchronous dynamics converges to a measure that is different from the Ising Gibbs measure. Therefore, an interesting question is whether these two measure are close with respect to a suitable metric. A result in this direction is obtained in [72]. The role of the *self-interaction* was indeed further investigated in order to derive an efficient method for approximating the sampling for the Ising stationary measure. In ([72], Equation (8)), a PCA dynamics was introduced via the *lifted Hamiltonian*

(35) H(σ,η)=−∑i,j∈Λi≠jJ¯ijσ(i)η(j)+∑i∈Λq(1−σ(i)η(i))

with ${\bar{J}}_{ij}$ as a positive symmetric matrix and q>0 as a positive parameter. The PCA dynamics is then the Markov chain defined by the transition probabilities

(36) $$p_{T,q}\left(\sigma ,\eta \right)=\frac{e^{-H(\sigma ,\eta )/T}}{\sum_{\zeta \in \Omega }e^{-H(\sigma ,\zeta )/T}}\;.$$

The parameter *q* can be interpreted as an inertial term: for large values of *q*, the dynamics is very slow, flipping few spins at each time. The transition probabilities (36) can be written as a product of single site updating probabilities, indeed,

(37) $$p_{T,q}\left(\sigma ,\eta \right)=\prod_{i\in \Lambda }{\bar{f}}_{T,\Theta_{i}\sigma }\left(\eta \left(i\right)\right)$$

with

(38) $${\bar{f}}_{T,\Theta_{i}\sigma }\left(\eta \left(i\right)\right)=\frac{\exp\left\{\frac{1}{T}\eta \left(i\right)\left[\sum_{j\in \Lambda \backslash \{i\}}{\bar{J}}_{ij}\sigma \left(j\right)+q\sigma \left(i\right)\right]\right\}}{2\cosh\left\{\frac{1}{T}\left[\sum_{j\in \Lambda \backslash \{i\}}{\bar{J}}_{ij}\sigma \left(j\right)+q\sigma \left(i\right)\right]\right\}}\;.$$

In [72], they posed for this model the question of Gibbsianess [73,74], and it was proved that the total variation distance between the invariant measure of the PCA defined in (36) and the Ising Gibbs measure goes to zero as the volume and the control parameter *q* goes to infinity. Notice that these approximation results do not apply directly to the model considered in the present review, which, indeed, is recovered for q=h=0 and ${\bar{J}}_{ij}=J_{ij}$. Afterwards, Refs. [75,76] extended the results of [72] also to the case of *weakly irreversible* PCA dynamics, and [77] beyond the high-temperature Dobrushin regime.

Finally, we mention the so-called *shaken* dynamics, which were introduced in [78] via a suitable lifted Hamiltonian, having only down–left interactions and depending on a parameter *q* that tunes the geometry of the system, which allows to interpolate between different lattices. For *q* large, the geometry is indeed the square lattice, and for finite *q*, the system lives on the hexagonal lattice, while for small *q*, the system becomes the product of independent one-dimensional lsing systems. Additionally, for these dynamics, the stationary measure tends to the Ising Gibbs measure in the thermodynamic limit.

## Data Availability

Not applicable.
